# Epithelial-Cell-Derived Extracellular Vesicles in Pathophysiology of Epithelial Injury and Repair in Chronic Rhinosinusitis: Connecting Immunology in Research Lab to Biomarkers in Clinics

**DOI:** 10.3390/ijms222111709

**Published:** 2021-10-28

**Authors:** Toru Takahashi, Robert P Schleimer

**Affiliations:** 1 Division of Allergy-Immunology, Department of Medicine, Northwestern University Feinberg School of Medicine, Chicago, IL 60611, USA; rpschleimer@northwestern.edu; 2 Department of Otolaryngology, Northwestern University Feinberg School of Medicine, Chicago, IL 60611, USA

**Keywords:** epithelial injury and repair, extracellular vesicles, microparticles, chronic rhinosinusitis, nasal polyps, EpCAM, E-cadherin, integrin β6, asthma

## Abstract

Epithelial barrier disruption and failure of epithelial repair by aberrant epithelial-mesenchymal transition (EMT)-induced basal cells observed in nasal mucosa of chronic rhinosinusitis (CRS) are speculated to play important roles in disease pathophysiology. Microparticles (MPs) are a type of extracellular vesicle (EV) released by budding or shedding from the plasma membrane of activated or apoptotic cells. MPs are detected in nasal lavage fluids (NLFs) and are now receiving attention as potential biomarkers to evaluate the degree of activation of immune cells and injury of structural cells in nasal mucosa of subjects with sinus disease. There are three types of epithelial-cell-derived MPs, which are defined by the expression of different epithelial specific markers on their surface: EpCAM, E-cadherin, and integrin β6 (ITGB6). When these markers are on MPs that are also carrying canonical EMT/mesenchymal markers (Snail (SNAI1); Slug (SNAI2); alpha-smooth muscle actin (αSMA, ACTA2)) or pro- and anti-coagulant molecules (tissue factor (TF); tissue plasminogen activator (tPA); plasminogen activator inhibitor-1 (PAI-1)), they provide insight as to the roles of epithelial activation for EMT or regulation of coagulation in the underlying disease. In this review, we discuss the potential of epithelial MPs as research tools to evaluate status of nasal mucosae of CRS patients in the lab, as well as biomarkers for management and treatment of CRS in the clinic.

## 1. Introduction

Chronic rhinosinusitis (CRS) is an inflammatory disease of the nose and paranasal sinuses. CRS causes profound decrements in quality of life and leads to over $10 billion of expense to the US healthcare system annually [1,2]. The respiratory epithelium functions in barrier defense from pathogens, exerts innate immune responses, and contributes to tissue repair [3]. Epithelial disruption and failure of repair have been reported in nasal mucosa of patients with CRS and are speculated to play important roles in the initiation and progression of disease [4,5,6,7]. Many inflammatory markers are relatively easily detected in nasal tissues by standard methodologies, such as ELISA, immunofluorescence/immunohistochemistry, or flow cytometry [8,9,10,11], and there is a strong desire to measure biomarkers in nasal secretions [12], as they are readily collected by lavage in an office or clinic setting and allow for frequent biomarker sampling. However, there are currently no assays reported to evaluate the status of epithelial injury and repair/remodeling without using tissue samples. Microparticles (MPs), one type of extracellular vesicle (EV), are detected in nasal lavage fluids (NLFs) and can be utilized as biomarkers to evaluate the degree of activation and injury of both immune and structural cells in nasal mucosae in subjects with sinus diseases, including CRS [13]. In this review, we focus on epithelial-cell-derived MPs, which can be detected by epithelial-specific markers using flow cytometry. We discuss their potential as research tools to evaluate the status of nasal mucosal inflammation in the lab, as well as use as biomarkers for management and treatment of CRS in the clinic.

### 1.1. Extracellular Vesicles (EVs)

EVs are nanoscale vesicles that are released from various types of cells. Although EVs have been overlooked as dust debris or noise for many years, growing evidence indicates that EVs are cell-derived particles that carry molecules from their parental cells and play roles in cell-to-cell communication [14,15,16,17,18,19,20]. Importantly, EVs have been detected in various human biofluids, such as blood, urine, saliva, breast milk, cerebrospinal fluids, sputum, bronchoalveolar lavage, and nasal lavage fluids [21,22,23,24], and have been found to have utility as biomarkers in various diseases. EVs have been classified into three main subtypes; exosomes, microparticles (MPs)/microvesicles, and apoptotic bodies [21]. Exosomes are smaller vesicles, with a diameter of around 100–200 nm, that are released from cells through exocytosis of multivesicular bodies. MPs are membrane vesicles with a diameter ranging from 100–1000 nm, which are released by budding or shedding from the plasma membrane of activated or apoptotic cells. Apoptotic bodies are yet larger vesicles, with diameters ranging from 1–5 um, that are released from fragmented apoptotic cells by blebbing of their plasma membrane. There are currently no specific markers to unequivocally distinguish these three EV subtypes, although there are distinguishing characteristics, such as size and composition. Consequently, EVs tend to be defined by physical characteristics such as size, defining small EVs (<200 nm) vs. large EVs (>300 nm), and immunochemical markers, such as CD41(+)CD31(+)EVs, CD63(+)EVs etc. [21]. In this review, when we quote results of EV data from publications, we use the terminology of EVs described in the cited papers without modification.

### 1.2. Microparticles (MPs) as Biomarkers for Human Diseases

**Figure 1:**
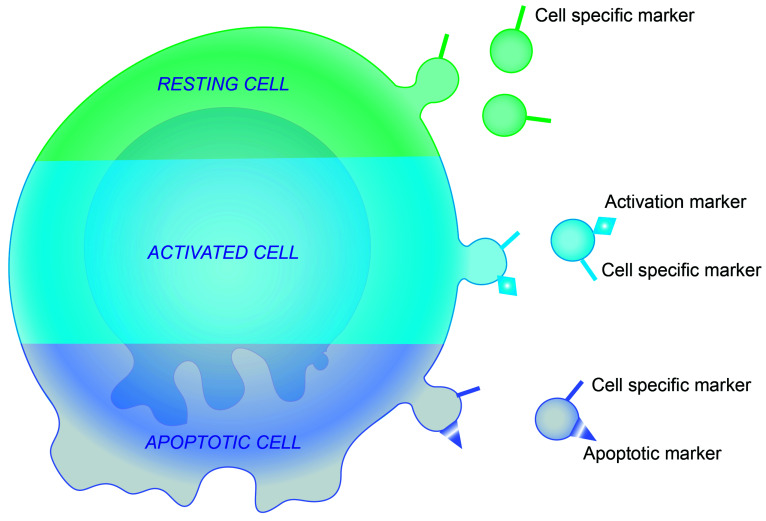
Schematic of MP release from activated and apoptotic cells. Adapted by permission from Elsevier. Ltd., Journal of Allergy and Clinical Immunology, Takahashi T, et al., copyright 5177010301838 [13].

Many papers have suggested that levels of MPs in human samples are potential biomarkers to evaluate the degree of activation or injury of structural and immune cells in a variety of diseases. Distinct from exosomes, which are released from cells through exocytosis of multivesicular bodies, MPs typically share the same cell surface membrane markers with their parent cells (Figure 1) [14,25]. For example, when endothelial cells are activated, E-selectin is upregulated and E-selectin-positive MPs are released. Thus, E-selectin(+)MPs reflect ongoing activation of endothelial cells. When apoptosis is induced in endothelial cells, Annexin V-positive, CD31 (endothelial membrane marker)-positive MPs are released. Thus, release of Annexin V(+)CD31(+)MPs reflects apoptosis of endothelial cells [26,27,28]. MP analyses have tremendous potential to evaluate the type of cells that are injured and the state of activation or apoptosis of injured cells directly in human samples collected from subjects under pathological conditions. Table 1 summarizes membrane markers and proteins detected in EVs of various cell origins. Levels of circulating endothelial MPs have been reported to be elevated in patients with vascular disorders, such as cardiovascular diseases, renal failure, and chronic obstructive pulmonary disease, where they have been correlated with progression, prognosis, and severe phenotypes of these diseases. They have also been utilized as new biomarkers to evaluate the degree of endothelial injury [29,30,31,32,33]. In addition, MPs detected in the blood, sputum, or bronchoalveolar lavage fluids (BALFs) have also been focused on as new biomarkers to evaluate condition of inflammation, disease severity, and progression in lung and airway diseases [34]. Circulating platelet MPs are increased in asthmatic patients when compared with non-asthmatic controls and are speculated to play a role in asthma pathophysiology. Tissue-factor-positive MPs are increased in the BALFs of interstitial lung disease patients and speculated to be involved in the pathophysiology [35]. Granulocyte-derived MPs are increased in the sputum of COPD patients and their levels are correlated with a worse performance index of COPD, such as the body mass index, airflow obstruction, dyspnea, and exercise capacity [24]. High levels of circulating leukocyte-derived MPs are reported to be associated with better outcomes in acute respiratory distress syndrome [36].

### 1.3. Current Methods to Enumerate EVs

The main obstacle of EV study is that gold standards are not yet available for isolation of EVs from biological samples and culture supernatants. EVs are highly heterogeneous in size and molecular compositions [15,74,75,76]. Cells release many other particles with characteristics overlapping with those of EVs and these molecules can actively interact with EVs on their surface [77]. In addition, components of EVs are significantly different among different types of parental cells and various human biofluids [78]. Therefore, although many methods to isolate EVs from various human biofluids and culture supernatants have been reported, such as ultracentrifugation, size exclusion chromatography, filtration-based methods, and immune-affinity-based methods [21], non-EV contaminants, such as protein complexes and other debris, are inevitably present in EV preparations and can interfere with results of EV analysis, especially “bulk EV measurement”, such as using Western blot and ELISA [76,79]. Thus, single-vesicle analysis, which gets information from individual EVs, is one of the most valuable approaches for the study of EVs [79]. The current preferred methods of single-EV analysis include nanoparticle tracking analysis (NTA), electron microscopy, and flow cytometry. NTA is a method for visualizing and measuring particles in liquids based on the rate of Brownian motion and is widely used to determine the particle size distribution of samples. By focusing a laser beam through a suspension of particles, the light scattered by each particle is recorded over disjointed time intervals by video to calculate the particle size distribution [80]. Image processing and recognition has been focused on as a promising field of the EV enumeration method in the future [81]. However, NTA collects data on all particles (both EVs and non-EV contaminants) in suspension, based on light scattering, and does not distinguish EVs from non-EV contaminants, making it difficult to enumerate EVs precisely [82]. Although electron microscopy provides meaningful information in terms of EV morphology, the morphology of EVs is heterogeneous among EV types and multi-preparation steps for electron microscopy can induce changes in their morphology [74,83]. Thus, it is necessary to label surface markers on EVs using immune-gold staining to distinguish EVs from non-EV contaminants. In addition, vesicles that contain significant numbers of non-EV contaminants can unevenly adhere on electron microscopy grids. Therefore, it is difficult to analyze heterogeneous EV populations and enumerate EVs precisely using electron microscopy [84]. Flow cytometry enables higher throughput of analysis of EVs based on membrane markers and their contents without EV isolation from samples [82,85].

### 1.4. Pitfalls of EV Analysis Using Flow Cytometry

**Figure 2:**
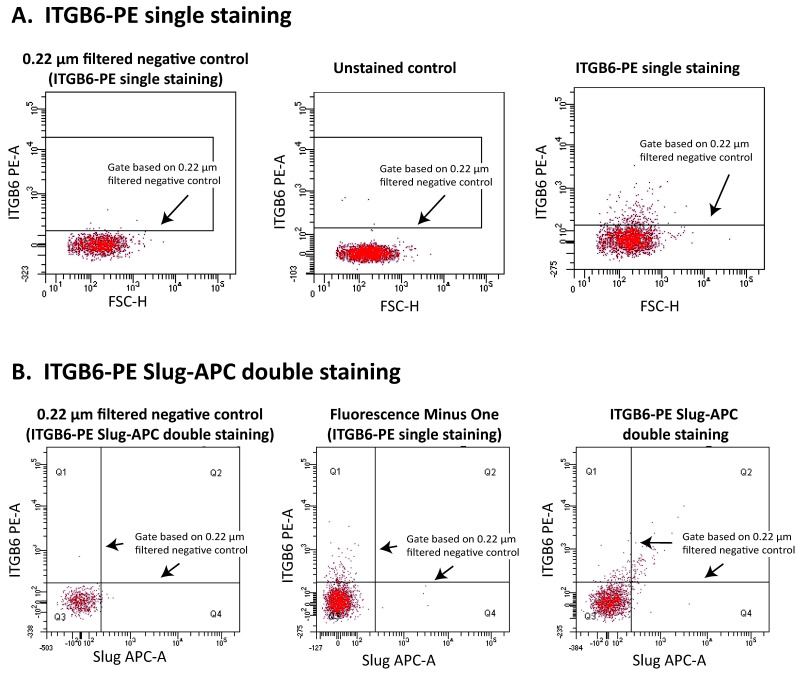
Unstained control, fluorescence minus one control, 0.22 μm filtered negative control. (**A**) Gating strategy of single ITGB6-PE(+)MPs in NLFs, (**B**) gating strategy of double slug-APC(+) ITGB6-PE(+) MPs in NLFs. Background noise is obviously included when the fluorescence-positive gate of ITGB6(+)MP subtypes were set according to unstained control or fluorescence minus one control. Thus, we used the 0.22 μm filtered negative samples to exclude background noise and set the fluorescence-positive gate of ITGB6(+)MP subtypes. Before the stained NLF samples were analyzed by flow cytometry, they were filtered using 0.22 μm membrane filters to exclude MPs in them. The filtered samples were used as negative MP controls when MPs were analyzed using the conventional flow cytometry, whose detection limit of particles was more than 0.3 μm. Adapted with permission from John Willey and Sons. Ltd., Allergy, Takahashi T, et al., 75, 3261–3289, copyright 5177010584585 [37].

Conventional flow cytometers were designed for the analysis of cells that are far larger than EVs. Thus, they are not easily adapted to EV measurement [86] and extensive flow cytometric expertise for sample preparation, acquisition, and data analysis is necessary for successful EV analysis [48,87]. For example, there are assay controls specific for EV analysis that are not usually used in cell analysis [86,88,89,90]. Sample buffer-only controls and sample buffer/staining reagent controls are important to distinguish background noise and false positive EV events due to unbound labels or aggregates of labels from true EV events. In addition, although fluorescence minus one (FMO) control and unstained control are also used to evaluate auto-fluorescence of samples and set compensation in EV measurement, they are sometimes not suitable to set fluorescence gates in the EV measurement (Figure 2). Furthermore, although the gates of EV analysis are often set using polystyrene beads, the shapes of the beads are different from shapes of EVs and a gating strategy based on the beads alone might be insufficient [86]. To address this point, we performed in vitro studies to isolate positive control EVs from culture supernatants of epithelial cells, human primary eosinophils, and human primary cultured mast cells and verified that epithelial EVs, eosinophil EVs, and mast cell EVs are properly detected in the gate setting based on polystyrene beads when we analyze these EV subtypes [13]. The minimal (not complete) framework for standardized reporting of EV flow cytometry experiments have been proposed by the International Society for Extracellular Vesicles (ISEV), International Society for Advancement of Cytometry (ISAC), and International Society on Thrombosis and Hemostasis (ISTH) [86].

### 1.5. Topology of EV Cargo (on the Surface or Inside of the EV Membrane?)

**Figure 3:**
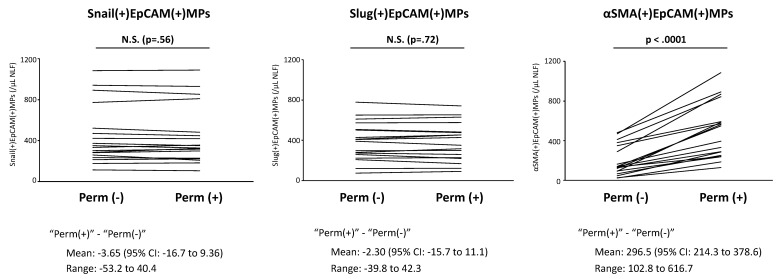
MP assay with and without permeabilization. We compared EpCAM(+)MPs using the MP assay either with or without permeabilization using NLFs collected from 18 subjects. Levels of Snail(+)EpCAM(+)MPs and Slug(+)EpCAM(+)MPs were not changed by permeabilization, although αSMA(+)EpCAM(+)MPs were increased by permeabilization (*p* < *0*.0001). Perm (+): MP protocol with permeabilization, Perm (−): MP protocol without permeabilization. “Perm(+)” − “Perm(−)”: difference in MP levels between the MP assay with and without permeabilization. Adapted with permission from John Willey and Sons. Ltd., Allergy, Takahashi T, et al., 75, 3261–3289, copyright 5177010584585 [37].

In addition to membrane markers, EVs have been found to carry intracellular proteins, nucleic acids, and organelles originating from the parent cells [16,17,18,19,20]. Analysis of EV contents can further enable their use in evaluation of the status (e.g., activation or apoptosis) of their cells of origin in clinical samples. It has been believed that intracellular proteins, RNA, and organelles in parental cells are incorporated inside EVs and encapsulation of cargo within EVs may protect them from degradation. However, recent studies have revealed that some intracellular proteins of the parental cells also exist on the surface of EVs [91]. For example, intracellular proteins, alpha-smooth muscle actin (αSMA, ACTA2), and cytokeratin have been reported to exist both inside EVs as well as on the surface of EVs [57,67,92,93,94]. In addition, DNA and chromatin have been detected on the surface of EVs [95,96,97,98] and transcription factors such as Snail (SNAI1) and Slug (SNAI2) might exist on the surface rather than inside MPs. Thus, it is important to compare EV assay results with and without permeabilization and clarify topology of EV cargo (i.e., whether the cargo is inside or on the surface of EVs) when analyzing their expression on EVs using flow cytometry [21]. We examined topology of αSMA, Snail, and Slug on EpCAM(+)MPs in NLFs by comparing results of a standard MP measurement protocol with and without the use of permeabilization using flow cytometry (Figure 3). Levels of Snail(+)EpCAM(+)MPs and Slug(+)EpCAM(+)MPs were not changed by permeabilization, suggesting surface expression. On the other hand, αSMA(+)EpCAM(+)MPs were significantly increased by permeabilization, suggesting internalized cargo. In these studies, we therefore elected to use permeabilization for detection of αSMA on epithelial MPs and omitted permeabilization for detection of Snail or Slug.

### 1.6. EV Functions in Tissue Injury, Repair, and Epithelial Barrier Defense

During the past 20 years, many studies have reported various functions of EVs in physiological and pathological processes. Generally, these functions are reported to depend on the surface molecules and/or contents of EVs, both of which are highly changed by the stimuli used to induce EV release [99]. There is no doubt that EVs play roles in cell-to-cell communication. However, due to the heterogeneity of EVs, as mentioned before, it is important to keep in mind that the results of these studies can be significantly influenced by EV isolation methods and non-EV contaminants in the EV preparations [21,78,99]. In addition, it remains to be determined how the majority of the reported EV functions casually contribute to physiological and pathological processes in vivo. Here, we summarize current understanding of EV functions related to tissue injury, tissue repair/remodeling, and epithelial barrier defense.

#### 1.6.1. Regulation of Inflammation

Epithelial-cell-derived EVs activate immune and structural cells, promote migration of immune cells, and induce proinflammatory cytokine release. For example, small EVs released from bronchial epithelial cells infected with human rhinoviruses induce release of IL-6, CCL5, and CXCL8 from macrophages and epithelial cells [100]. EVs released from epithelial cells exposed to hypoxia encapsulate caspase-3, activate macrophages via the ROCK-1 pathway, and induce secretion of pro-inflammatory cytokines [101]. Exosomes released from IL-13-stimulated epithelial cells enhance proliferation and chemotaxis of macrophages, while exosomes released from unstimulated epithelial cells do not have these effects [102].

EVs released from other cell types are also reported to have pro-inflammatory properties. MPs released from phorbol myristate acetate (PMA)-activated T cells can induce degranulation and production of IL-8, oncostatin M, and IL-24 release by mast cells [103]. Another study reported that PMA-stimulated T cell-derived MPs also induce the production of TNF-α and IL-1β in human monocytes [104]. Finally, MPs released from actinomycin D-stimulated T cells induce production of the proinflammatory cytokines TNF-α, IL-6, and IL-8 and trigger apoptosis with upregulation of caspase-3, -8, and -9 in bronchial epithelial cells [105]. Macrophages challenged with LPS release EVs carrying damage-associated molecular patterns (DAMPs), such as histones on their surface, which are speculated to contribute to lethal systemic inflammation in patients with sepsis [63,106]. Antigen-presenting cells are reported to release EVs carrying major histocompatibility complex (MHC)–peptide complexes that can promote immune responses. EVs released from dendritic cells are reported to transfer functional MHC–peptide complexes to other dendritic cells that activate T cells [53,69]. EVs released from follicular B cells contact the surface of follicular dendritic cells through their surface molecules, such as phosphatidylserine, CD11a, and C3-derived fragments, and convey MHC class II to the dendritic cells [69].

EVs released from some cell types have been reported to have both pro-and anti-inflammatory effects. For example, neutrophil MPs have been reported to deliver active myeloperoxidase to injured mucosa to inhibit epithelial wound healing [56]. On the other hand, neutrophil MPs have been reported to contain the anti-inflammatory protein annexin 1 and display inhibitory properties on target cells [107]. Platelet MPs are reported to be a source of IL-1, IL-6, and tumor necrosis factor-α (TNF-α) and amplify inflammation in arthritis [48]. On the other hand, platelet MPs have also been reported to contain 12-lipoxygenase (12-LO) protein and act as mediators in transferring 12-LO to mast cells, leading to the production of lipoxin A4 by mast cells and attenuating inflammation [49]. Furthermore, mast cells release EVs carrying IgE on their surface and have been shown to attenuate or stimulate allergic responses via interaction between IgE receptors on the surface of EVs and immune complex/IgE according to activation status of mast cells. For example, EVs released from unstimulated mast cells bind to free IgE in the circulation and attenuate allergic responses [59]. On the other hand, EVs released from IgE/antigen-stimulated mast cells can enhance allergic responses by facilitating interaction of EV-associated antigens with FcεRI-bound IgE on mast cells, promoting EV uptake via endocytosis and delivering antigen to the mast cells [58].

Stem-cell-derived EVs have been reported to have anti-inflammatory effects, especially through transfer of mitochondria. For example, in central nervous system disorders such as multiple sclerosis, neural stem cells transfer functional mitochondria via EVs into inflammatory mononuclear phagocytes, restoring normal mitochondrial functions and cellular metabolism of the phagocytes, and downregulate pro-inflammatory phagocyte markers [108]. Mesenchymal stem-cell-derived EVs also transfer mitochondria to macrophages and enhance oxidative phosphorylation, which can suppress cytokine production, increase M2 macrophage marker expression, and augment the phagocytic capacity of macrophages in the context of acute respiratory distress syndrome (ARDS) [19]. These anti-inflammatory effects of stem-cell-derived EVs are speculated to play roles in resolution of inflammation and repair of damaged tissue under various pathological conditions.

#### 1.6.2. Extracellular Matrix (ECM) Interactions and Remodeling

EVs interact directly with the extracellular matrix (ECM) or cells associated with the ECM, controlling production of cytokines, proteases, and glycosidases, and are speculated to modulate tissue repair and remodeling [109,110]. B cell-derived exosomes express functional integrin β1 and β2, which can bind to collagen-I, fibronectin, and activated fibroblasts [70]. Exosomes released from reticulocytes during their maturation were reported to bind to fibronectin via integrin α4β1 [111]. In addition, exosomes released from hypoxic renal tubular epithelial cells were reported to contain TGF-β and activate fibroblasts for increased collagen-1 production [112]. MPs released from activated or apoptotic T cells strongly induce the synthesis of matrix metalloproteinases (MMP)-1, MMP-3, MMP-9, and MMP-13 in fibroblasts [113]. LPS-stimulated monocyte-derived EVs induce cytokine secretion and upregulation of MMP gene expression in mesenchymal stem cells and facilitate tissue remodeling [114]. Furthermore, glycosidases such as sialic acid and heparanase have also been detected on the surface of exosomes [115]. LPS-stimulated microglial cells release small EVs carrying neuraminidase-1, which can trigger the release of neurotrophin from the cells and protect brain cells through extracellular matrix remodeling [116,117].

#### 1.6.3. Angiogenesis

The creation of a new vasculature via angiogenesis is an essential process for normal tissue repair [118]. Many studies have reported that EVs, especially those released from endothelial cells, endothelial progenitor cells, platelets, leukocytes, erythrocytes, and cancer cells, modulate angiogenesis through EV content and surface protein expression in normal tissue repair processes and under various pathological conditions [99]. A recent study reported enhancement of angiogenesis by αvβ6-integrin-positive cancer-cell-derived EVs. Integrin αvβ6 is known to be upregulated in various types of cancers and its expression negatively correlates with the survival time of patients [119]. Prostate cancer cells release small EVs expressing αvβ6 integrin and transfer αvβ6 integrin to immortalized human dermal microvascular endothelial cells, enhancing angiogenesis [120].

#### 1.6.4. Epithelial–Mesenchymal Transition (EMT)

EMT is a process during which epithelial cells acquire mesenchymal features [6,121,122,123] and is speculated to be an essential process in normal epithelial repair and to have involvement in pathological airway remodeling [122,123]. Many studies reported that EVs, especially cancer-cell-derived EVs, contain EMT-regulating molecules and induce EMT in host cells [124]. For example, colorectal-carcinoma-derived EVs contain Wnt1 and enhance colorectal carcinoma cell proliferation and migration through Wnt/β-catenin signaling, a pathway associated with EMT activation [125]. EVs released from cancer-associated fibroblasts carried Snail and further promote EMT in the A549 type II pulmonary epithelial cell line [126]. EVs released from pancreatic ductal adenocarcinoma carry tenascin-C and promote further EMT of the cell line [127]. In addition to cancer-cell-derived EVs, EVs released from HMC-1 mast cell lines have also been reported to contain TGF-β1 and induce EMT in epithelial cells [61].

#### 1.6.5. Regulation of Senescence and Induction of Pathological Fibrosis

Senescence is induced in fibroblasts and controls fibrotic pathways necessary for normal tissue repair [128]. Accumulation of senescent epithelial cells has also been observed in fibrotic disorders in lung and kidney and accelerated senescence is speculated to be crucial for the initiation and progression of fibrosis in these diseases [128,129]. EVs are reported to be involved in regulation of cellular senescence. Small EVs isolated from young human donor fibroblasts have more intrinsic glutathione-S-transferase activity than those from elderly human donors and ameliorate senescence-related tissue damage [130]. In addition, EVs carrying extracellular nicotinamide phosphoribosyltransferase (eNAMPT) have been detected in the circulating blood in humans and mice and promote systemic NAD+ biosynthesis and counteract aging [131]. Furthermore, EVs are reported to facilitate fibrosis through induction of senescence in fibroblasts and epithelial cells. EVs released from senescent fibroblasts contain the interferon pathway protein IFITM3 and transmit senescence to normal cells [132]. EVs released from fibroblasts in the lung of idiopathic pulmonary fibrosis contain WNT-5α, induce lung cell senescence, and contribute to progression of fibrosis [133]. EVs released from hypoxic injured proximal tubular cells contain TGF-β1, an inducer of senescence that can also activate fibroblasts to initiate fibrosis [112].

#### 1.6.6. Regulation of the Coagulation Cascade

The interaction between the coagulation system and inflammation plays an important role in tissue repair [134,135,136,137]. In response to injury, the coagulation system is activated, followed by activation of the fibrinolytic system. Abnormal activation of the coagulation cascade and dysfunction of the fibrinolytic cascade are speculated to result in failure of tissue repair, such as that which occurs in thrombotic disorders associated with abnormal tissue remodeling. Many studies have demonstrated that EVs carry activators of the clotting cascade on their membrane and exhibit procoagulant properties in physiological and pathological processes. For example, endothelial MPs carry von Willebrand factor multimers, which promote platelet aggregates and increase the stability of the aggregates thus formed [43]. Phosphatidylserine (PS) on the surface of circulating MPs from platelets, leukocytes, erythrocytes, and endothelial cells are reported to bind to coagulation factors, such as factor IXa, Va, Xa, and VIII, and induce procoagulant activity, promoting thrombin formation and activation in vitro [44]. In addition, PS-positive MPs are significantly increased in the circulation of septic patients when compared with healthy controls and are speculated to play roles in augmenting coagulation in sepsis [44]. Human saliva and urine contain abundant tissue factor (TF)-positive EVs with functional procoagulant properties that can contribute to host defense of injured epithelium by reducing blood loss and preventing invasion of microorganisms [23]. TF-positive epithelial MPs with procoagulant activity are released from alveolar epithelial cells stimulated with proinflammatory cytokines in vitro, increased in bronchoalveolar lavage (BAL) fluids collected from patients with ARDS, and are speculated to contribute to fibrin deposition in the air space in the pathophysiology of ARDS [138,139]. Thrombotic events occur in up to one-third of patients with COVID-19 and are associated with increasing disease severity and worse clinical outcomes [140]. Circulating TF-positive EVs were also increased in patients with COVID-19, which is associated with severity and mortality [141].

Although EVs have been mostly reported to have procoagulant properties, increasing numbers of studies have recently reported that EVs can carry anticoagulant molecules on their surface and have anti-coagulant/fibrinolytic properties [142,143]. For example, circulating endothelial MPs express tissue plasminogen activator (tPA) and tPA/inhibitor complexes and circulating leukocyte-derived MPs express urokinase-type plasminogen activator (uPA) and its receptor (uPAR). Plasminogen is activated into plasmin on the surface of these MPs in vitro [45,46]. Erythrocyte-derived MPs can bind protein S on their surface and modulate anticoagulant actions of the protein C system in vitro [72]. Circulating endothelial MPs carrying endothelial protein C receptor and thrombomodulin have been detected in circulating blood of severe sepsis patients and were further elevated with the progression of sepsis-induced disseminated intravascular coagulation [47].

#### 1.6.7. Antimicrobial and Antiviral Functions

Some populations of EVs have been reported to have antibacterial or antiviral functions and speculated to protect the epithelial barrier against bacterial or viral pathogens. Neutrophil MPs released in response to stimulation of opsonized particles have the capacity to impair bacterial growth [57]. Interestingly, this antibacterial effect was not observed in neutrophil MPs either released spontaneously or released in response to PMA stimulation. In addition, urinary exosomes of healthy individuals released from renal tubular epithelia are enriched with innate immune proteins and induce bacterial lysis and inhibit bacterial growth in vitro [144]. Exosome-like vesicles with antiviral properties are also released from air–liquid interface cultured human bronchial epithelial cell cultures into the apical secretions [145]. Furthermore, the bitter taste receptor family, a class of G protein-coupled receptors (GPCRs), is expressed on epithelial cells and epithelial cells activated with bitter taste ligands enhance antibacterial effects through increased secretion of β-defensin and increased ciliary beat frequency [146,147]. GPCR families, including the bitter taste receptor family, have been detected on EVs [148]. GPCRs on the surface of EVs are speculated to control epithelial barrier defense through horizontal transfer of functional receptors between epithelial cells [149].

### 1.7. Epithelial Barrier Disruption and Abnormal Epithelial Repair in CRS

CRS is commonly divided into a phenotype with nasal polyps (CRSwNP) and a phenotype without nasal polyps (CRSsNP). Detachment of epithelial cells from the basal membrane due to inflammation was observed in nasal mucosa of both CRS phenotypes [150]. In addition, decreased expression of junctional proteins in the apical epithelial junctional complex (tight junction and adherens junction) has also been observed [151]. These observations led to the hypothesis that deficiencies in epithelial barrier function might compromise the interaction between the host and external immune stimuli and play important roles in pathophysiology of CRS [5]. Furthermore, basal cells are populations of undifferentiated epithelial cells that play important roles in the repair of damaged epithelium [7,152,153,154]. Tissue-based studies recently reported that basal cell hyperplasia was observed [7], with evidence of EMT, in nasal epithelium spanning the mature polyp itself, which is often either acanthotic or consists of only a single layer of basal cells [6] and is more often observed in the epithelium on the polyp perimeter where acanthosis is frequently observed [155]. In addition, fibrin deposition was detected locally in nasal polyps [156]. These reports also led to the hypothesis that epithelial repair fails due to abnormal basal cell function with aberrant EMT and coagulation cascade activation, which might causally contribute to abnormal tissue remodeling, especially polyp formation, in CRS. However, these pathological lesions are unevenly distributed in tissue and are difficult to quantify [10,157]. In addition, biopsy of nasal tissues is invasive and not generally practical in the clinic [158]. A new assay able to evaluate the status of epithelial repair and remodeling in NLFs would further facilitate non-invasive assessment of sinus disease in outpatients in the clinic.

### 1.8. MPs in Nasal Lavage Fluids as Biomarkers for Chronic Rhinosinusitis

Although levels of inflammatory mediators are often too low to evaluate in nasal secretion [12], we succeeded in detecting MPs released from immune cells and structural cells in NLFs collected from controls and subjects with CRS using flow cytometry [13]. Table 2 is the list of MP subtypes detected in NLFs. MPs are usually detected by surface markers on MPs using antibodies. Thus, the smaller the size of the target vesicles, the more difficult to distinguish true MP events from false positive MP events due to non-specific binding of antibodies to non-MP particles. Due to the fact that recent studies have reported that the surface of an EV can interact with various molecules actively and is very sticky, as mentioned earlier, we did not seek to measure small EVs (<300 nm), and defined MPs as large EVs (>500 nm) with cell membrane markers (private discussion with the application support group in BD Biosciences). In addition, MP levels in human biofluid samples are usually evaluated as a concentration of target MPs in the biofluid samples [86] and not standardized by total protein levels of the samples because the total protein levels in biological samples are significantly influenced by non-MP contaminants (e.g., lipoprotein, albumin, and other plasma proteins coming from circulating blood are detected in NLFs in response to epithelial and endothelial disruption). Thus, MP levels in NLFs were evaluated as target MP counts per 1 micro-liter NLFs.

### 1.9. Epithelial Injury, Basal Cell Activation, and Epithelial MPs in CRS

**Figure 4:**
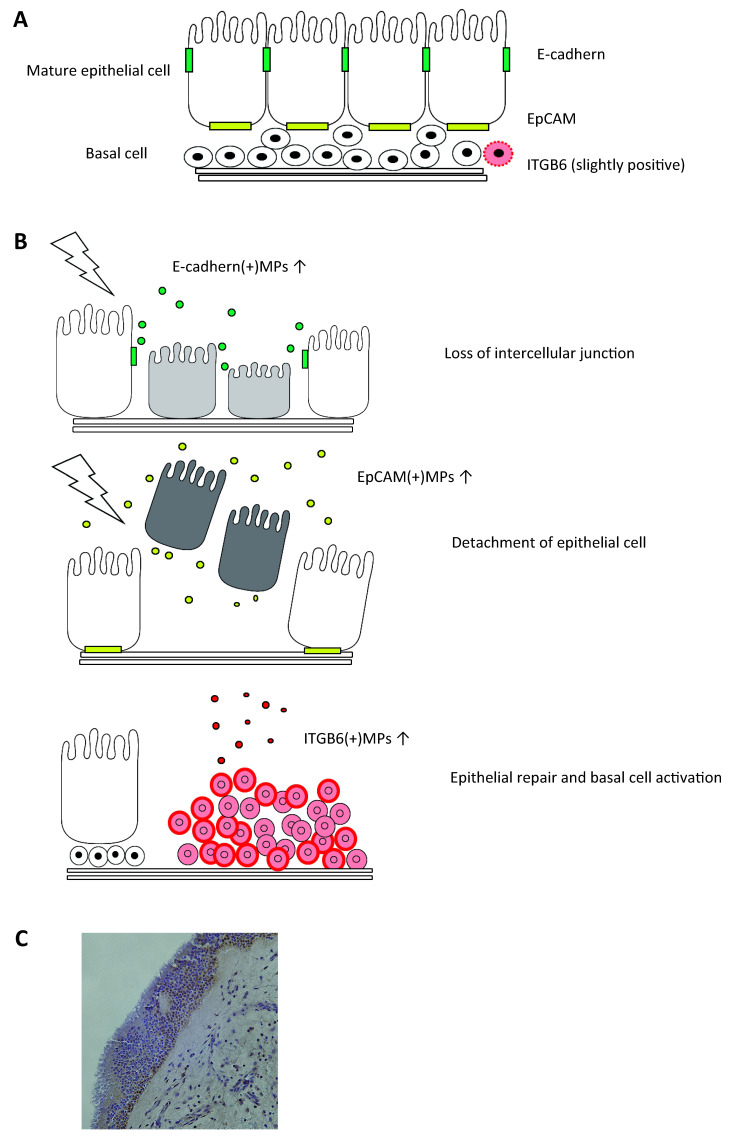
Location of epithelial markers and epithelial MP release. (**A**) Location of E-cadherin, EpCAM, and ITGB6. (**B**) Schematic of release of E-cadherin(+)MPs, EpCAM(+)MPs, and ITGB6(+)MPs. (**C**) Immunohistochemistry for ITGB6 in sinonasal uncinated tissue from CRSwNP. A representative image under 40× magnification is shown. ITGB6 was positive on hyperplastic basal cells on injured epithelium, perhaps indicative of repair.

Three types of MPs expressing epithelial-specific markers, EpCAM(+)MPs, E-cadherin(+)MPs, and Integrin β6 (ITGB6)(+)MPs, were measured in NLFs to compare status of epithelial injury and repair among controls, CRSsNP, CRSwNP, and aspirin-exacerbated respiratory disease (AERD), a severe form of CRSwNP with history of aspirin hypersensitivity, difficult to control comorbid asthma, and frequently recurrent polyposis after surgery [37]. EpCAM and E-cadherin are constitutively expressed on the basolateral side or adherens junctions of epithelial cells, respectively [159]. Thus, the release of E-cadherin(+)MPs in the nasal cavity may reflect a loss of intercellular adherens junctions and the increased EpCAM(+)MPs may reflect detachment of epithelial cells from basement membranes [13] (Figure 4). ITGB6 is an epithelial-specific adhesion molecule that is rarely expressed on normal epithelium but is upregulated on basal cells during epithelial repair [160,161,162]. ITGB6 is also upregulated during EMT, in contrast to the majority of epithelial markers such as EpCAM and E-cadherin that are downregulated [121,163,164]. ITGB6(+)MPs are released from A549 cells stimulated with TGF-β, although neither E-cadherin nor EpCAM(+)MPs are released [37]. Therefore, ITGB6(+)MP release may better reflect ongoing basal cell activation during repair, rather than epithelial injury (Figure 4).

**Figure 5:**
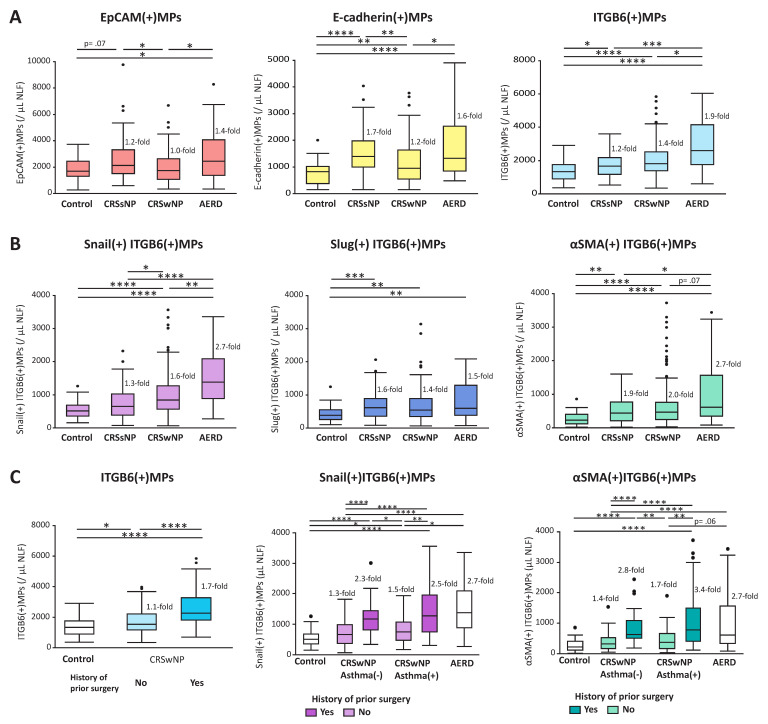
Comparisons of epithelial MP levels in NLFs. (**A**) Comparison of EpCAM(+)MPs, E-cadherin(+)MPs, and ITGB6(+)MPs among control, CRSsNP, CRSwNP, and AERD. (**B**) Comparisons of dual-positive EMT marker (Snail, Slug, or αSMA) ITGB6(+)MPs among control, CRSsNP, CRSwNP, and AERD. (**C**) Comparison of ITGB6(+)MPs between CRSwNP with history of prior surgery (cases undergoing surgery to treat polyp recurrence) vs. without prior surgery (primary surgical cases), and comparisons of Snail(+) ITGB6(+)MPs and αSMA(+) ITGB6(+)MPs between CRSwNP with asthma vs. without asthma (including those with history of prior surgery and those without prior surgery, as indicated). We enrolled cases with CRSwNP (*n* = 196), CRSsNP (*n* = 70), and AERD (*n* = 31) who had endoscopic sinus surgery (ESS) at Northwestern Memorial Hospital. In addition, we also enrolled control cases (*n* = 47) who underwent surgery other than ESS without sinus disease. NLFs were collected at the time of surgery, and MPs were measured using a BD FACS LSRII flow cytometer (BD Biosciences, Erembodegem, Belgium). *, *p* < 0.05; **, *p* < 0.01; ***, *p* < 0.001; ****, *p* < 0.0001; ##-fold, significantly higher by ##-fold vs. control. Adapted with permission from John Willey and Sons. Ltd., Allergy, Takahashi T, et al., 75, 3261–3289, copyright 5177010584585.

We reported that the increase of epithelial MP subtypes varied according to the phenotype of CRS (Figure 5A) [37]. Levels of E-cadherin(+)MPs and EpCAM(+)MPs were higher in CRSsNP and AERD than in CRSwNP. Interestingly, although E-cadherin(+)MPs were significantly increased in subjects with CRSwNP when compared with controls, EpCAM(+)MPs were not increased. Eosinophilic type 2 inflammation is one of the most important features in the pathophysiology of CRSwNP [9], and type 2 cytokines have been reported to attenuate expression of junctional proteins in apical junctional complex on air–liquid-cultured epithelia in vitro [165]. Based on the MP levels in NLFs, a high degree of epithelial injury with detachment of epithelial cells from basement membrane occurs in the nasal mucosa of CRSsNP and AERD, whereas a lower degree of epithelial injury focused on disruption of intercellular junctions is the main feature of epithelial disruption in CRSwNP. On the other hand, ITGB6(+)MPs were higher in polypoid CRS phenotypes, CRSwNP and AERD, than in the non-polyploid phenotype, CRSsNP (Figure 5A). In addition, among CRSwNP cases, high ITGB6(+)MP levels were associated with those with history of prior surgery (Figure 5C). Basal cells may be more highly activated during epithelial repair in the nasal mucosa of patients with polypoid disease than in patients with non-polypoid CRS, especially in nasal epithelium of patients with recurrent CRSwNP.

### 1.10. Epithelial Mesenchymal Transition and Epithelial MPs in CRS

We examined Snail (SNAI 1), Slug (SNAI 2), and alpha-smooth muscle actin (αSMA, ACTA2) carried on ITGB6(+)MPs to evaluate the status of basal cells undergoing EMT in nasal mucosa of CRS [37]. Snail and Slug are EMT-promoting transcription factors that initiate EMT [166]. αSMA is also upregulated during EMT and often used as a biomarker of the process [6,121]. These three EMT MPs are released from EMT-induced A549 cells stimulated by TGF-β in vitro [37]. In addition, levels of αSMA(+)ITGB6(+)MPs in NLFs were lower than levels of Snail(+)ITGB6(+)MPs (*p* < 0.0001) or Slug(+)ITGB6(+)MPs (*p* < 0.0001), which may reflect the complex relationships between EMT-activating transcription factors and mesenchymal markers upregulated in epithelial cells during EMT.

Elevated counts of EMT-induced epithelial cells have been detected by standard immunohistochemistry in the sinonasal epithelium of both CRSsNP and CRSwNP [11]. We reported that three dual EMT-marker-positive ITGB6(+)MPs were significantly elevated in CRSsNP, CRSwNP, and AERD when compared with controls (Figure 5B). In addition, dual Snail(+)ITGB6(+)MPs were significantly higher in polypoid phenotypes CRSwNP and AERD than in CRSsNP, in spite of there being no difference in Slug(+)ITGB6(+)MP levels. Although they are both in the SNAI family, Snail and Slug differ in their respective target genes and expression in tissues [167,168,169], suggesting qualitatively different roles in the EMT process between CRSsNP and polypoid phenotypes. However, there was no relationship between levels of ITGB6(+)MP subtypes and clinical features in CRSsNP, (e.g., a history of prior sinus surgery, asthma, or atopy comorbidity, or symptoms such as facial or ear pain). On the other hand, all three of these ITGB6(+)MP subtypes, especially Snail(+)ITGB6(+)MPs and αSMA(+)ITGB6(+)MPs, were significantly higher in CRSwNP cases with history of prior surgery than those without prior surgery, independent of asthma status (Figure 5C). Highly elevated levels of ITGB6(+)MPs, which carry Snail or αSMA in patients with CRSwNP and a history of prior surgery, may indicate aberrant or excessive EMT activation and epithelial repair in those severe CRSwNP phenotypes. Although CRSwNP subjects with comorbid asthma have a higher recurrence rate after surgery than those without asthma and are regarded as a severe phenotype with high type 2 inflammation [9,158,170,171], there were weak correlations between levels of ITGB6(+)MP subtypes and eosinophil cationic protein (ECP) [37], indicating that the pattern of epithelial repair and EMT indicated by ITGB6(+)MP subtypes is distinct from that of type 2 inflammation. Abnormal basal cell activation with aberrant EMT could be a new target of therapeutic strategy to prevent polyp recurrence in CRS.

### 1.11. Coagulation Cascade and Epithelial MPs in CRS

Tissue factor (TF) is rapidly upregulated at the site of tissue injury, activates the extrinsic pathway of the coagulation system, and plays an important role in linking early inflammatory responses and subsequent tissue repair processes [136]. In addition, TF itself is reported to promote wound repair of local airways [172]. Plasminogen activator and plasminogen activator inhibitor 1 (PAI-1) are expressed by injured epithelia, and activation of plasmin formation by plasminogen activation induces extracellular proteolysis of fibrin clots in injured tissues and is an important step in normal tissue repair [137]. In addition, the proliferation of a granular tissue is observed during early stages of polyp formation [155], and the expression of tPA and PAI-1 is abundant in cells of granulation tissue in well-granulating wounds, but not in the granulation tissue cells of nonhealing ulcers [137]. We succeeded in detecting TF, tPA, and PAI-1 on epithelial MPs in NLFs and started to perform preliminary studies to compare expression of TF, tPA, and PAI-1 on epithelial MPs in NLFs between CRSsNP and CRSwNP to evaluate patterns of activation of the coagulation and fibrinolysis cascades in response to epithelial injury. An important point is that tPA and PAI-1 were detected on the surface of epithelial MPs and it is difficult to clarify where these molecules originated because they are not membrane proteins, unlike TF. These molecules interact actively on the surface of EVs, as we mentioned earlier. Thus, their expression pattern on EVs may indicate presence of the fibrinolytic cascade in the vicinity of EVs.

### 1.12. Challenges and Future Directions of Epithelial MPs in NLFs

**Figure 6:**
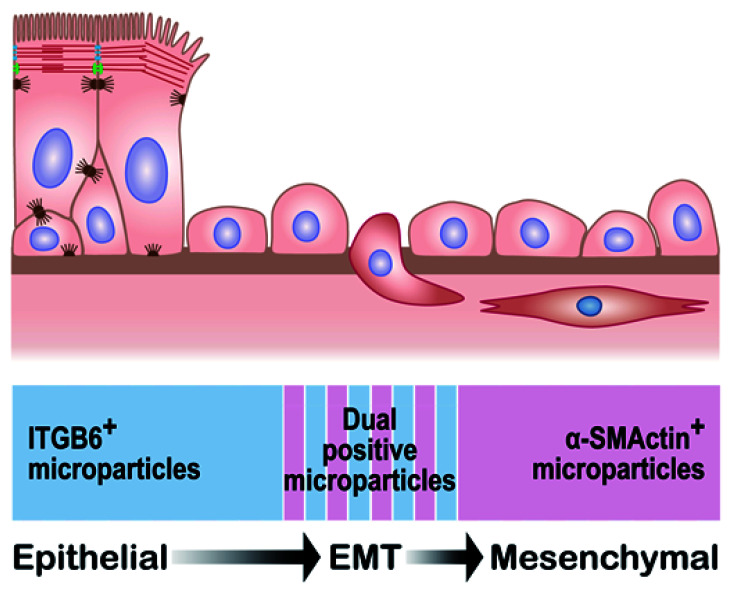
Hypothetical model of MP detection of basal cells/EMT. Differentiated epithelial cells (top left) transition to mesenchymal cells (lower right). During that process, the ITGB6-positive epithelial cells lose the epithelial marker and begin to express the mesenchymal marker αSMA (bottom). According to the hypothesis, dual-positive MPs are released only by cells that simultaneously express both markers (striped area, bottom).

There is no currently accepted EMT marker that is specific and persistent throughout the EMT process. It has been reported that epithelial cells pass through intermediate states of EMT, during which they can manifest both epithelial and mesenchymal traits (partial EMT) after injury [173,174]. This was part of the basis of the rationale to use dual-positive EMT marker ITGB6(+)MPs to evaluate the status of EMT; dual EMT-marker-positive ITGB6(+)MPs are speculated to be released only by cells that simultaneously express both ITGB6 and EMT markers during a transitional state (Figure 6). However, although ITGB6 is upregulated during EMT, the majority of mesenchymal cells were ITGB6-negative in nasal tissues from CRS subjects analyzed by immunohistochemistry (Figure 4C). Many or most transitional cells might completely lose some epithelial markers, such as ITGB6, during EMT in CRS. In addition, although ITGB6 upregulation after injury reverts to a low level after completion of epithelial repair, EMT may still continue, even after downregulation of ITGB6, due to failure of epithelial repair in CRS. We also discovered αSMA(+)EpCAM(+)MPs. However, correlation of αSMA(+)EpCAM(+)MPs with CRSwNP cases with prior surgery was significantly lower than those employing ITGB6(+)MPs. Additional studies comparing biopsied tissue-based and MP-based evaluation of EMT would be of significant value in validating the extent to which levels of EMT-related MP subtypes reflect the ongoing process in vivo. Due to the fact that the MP assay in NLFs is representative of the condition in the entire nasal cavity, it would also be of interest to collect MPs released from nasal tissues selected for biopsy using a sponge-based approach (placing a small sponge on target nasal mucosa to collect nasal secretion from the mucosa) in order to directly compare standard immunohistochemical methods to the MP approach [175]. Furthermore, as mentioned above, the perimeter of polyps appears to have the highest level of acanthosis [155] and is speculated to be a main source of ITGB6(+)MPs in NLFs. To examine whether ITGB6(+)MPs originate from nasal epithelium on the perimeter of nasal polyps using the sponge-based approach is worthy of further study. Finally, because NLF collection is less invasive than biopsy of nasal mucosa and easy to perform in the clinic, it is possible to follow levels of ITGB6(+)MP subtypes with subsequent measurements in a longitudinal study. It is important to see whether these ITGB6(+)MP levels are persistently elevated or not to confirm the assignment of activated basal cell endotypes in CRSwNP subjects with a history of prior surgery. Similarly, it would be of interest to monitor MPs that are indicative of the EMT process before, during, and after therapeutic interventions (e.g., with glucocorticoids or biologics that disrupt type 2 inflammatory processes) to evaluate whether these drugs improve barrier function by halting the persistent EMT process that has been observed.

## 2. Conclusions

In this review, we proposed the potential use of MPs in NLFs as an invaluable tool in the study of CRS in the laboratory and as new biomarkers for CRS in the clinic, using epithelial MPs as an example (Table 3). By comparing subtypes of epithelial MPs expressing different epithelial markers and protein cargo, we can detect differences in the degree and status of epithelial injury and repair among CRSsNP, CRSwNP, and AERD. Furthermore, we found that abnormal basal cell activation with aberrant EMT is connected to recurrent polyposis in CRSwNP, and controlling basal cell activation might be beneficial as a therapy to prevent polyp recurrence in CRS. Due to the fact that NLFs are readily collected in an office or clinic setting, it is possible to perform a longitudinal study with multiple time points of sample collection to follow the condition of nasal mucosa in response to disease progression and treatment. We believe that MPs in NLFs may help to provide new insights into the pathophysiology of CRS and play a valuable role in testing the link between various findings detected in nasal tissue samples in the lab and the natural history, comorbidities, and long-term outcomes of CRS in the clinic.

## Figures and Tables

**Table 1:** Membrane markers and intracellular proteins detected in EVs of various cell origins.

Origin	Membrane Marker	Intracellular Protein, Proteins Interacting with EVs	References
Epithelial cell	EpCAM, E-cadherin, Integrin β6 (ITGB6)	tPA, PAI-1, TF, Snail, Slug, αSMA, TGF- β	[13,35,37,38,39]
Endothelial cell	CD31, CD51, CD54, CD62E, CD105, CD144, CD146, VCAM-1, CD309 (VEGF-R), ICAM-1	ACE, vWF, TF, coagulation factors (factor IXa, Va, Xa, and VIII, tPA, endothelial protein C receptor, thrombomodulin)	[13,29,30,31,40,41,42,43,44,45,46,47]
Platelet	CD31, CD41, CD42a,b,d, CD61, CD62P (P-selectin), CD63	TF, CD63, NF-kB, IL-1, IL-6, TNF-α, lipoxygenase 12 (12-LO) protein, coagulation factors (factor IXa, Va, Xa, and VIII)	[13,30,40,44,48,49,50]
Leukocyte	CD11a,b, CD13, CD14, CD16, CD31, CD45, CD62L (L-selectin), ICAM-1	TF, Complement C3, MMPs, ICAM-1, coagulation factors (factor IXa, Va, Xa, and VIII), uPA), and its receptor (uPAR)	[44,45,46,51,52,53,54]
Eosinophil	EMR1, CD66b, CD69	CD63, EPO, MBP, ECP	[13,55]
Neutrophil	CD66b, CD11b, CD177, FcγRIII	CD63, CD81, CD82, CD9, IL-1β, annexin 1, MPO	[48,56,57]
Mast cell	CD117 (c-kit), FcεRI	Tryptase, CD63, IgE, TGF-β1, CD137	[13,58,59,60,61]
Basophil	CD203c	CD63	[13]
Monocyte	CD14, CD11c		[62]
Macrophage	CD206, CD71	DAMP (histone)	[63]
T cell	CD4, CD8		[64,65,66,67]
B cell	CD19, CD20	C3-derived fragments, MHC class II, integrin β1, integrin β2	[65,68,69,70]
Erythrocyte	CD235a (glycophorin-A), CD238	TF, protein S	[67,71,72,73]

tPA, tissue plasminogen activator; PAI-1, plasminogen activator inhibitor 1; TF, tissue factor; Snail, SNAI1; Slug, SNAI2; αSMA, alpha-smooth muscle actin (ACTA2); ACE, angiotensin-converting enzyme; vWF, von Willebrand factor; MMP, matrix metalloproteinase; EMR1, EGF-like module-containing mucin-like hormone receptor-like 1; EPO, eosinophil peroxidase; MBP, major basic protein; ECP, eosinophil cationic protein; FcγRIII, low-affinity IgG Fc receptor; MPO, neutrophil myeloperoxidase; FcεRI, high-affinity receptor for the Fc region of IgE; DAMP, damage-associated molecular patterns.

**Table 2:** MP subtypes detected in NLFs.

MP Type	Definition
Endothelial MPs	CD62e(+)MPs CD31(+)CD41(−)MPs CD144(+)MPs
Epithelial MPs	EpCAM(+)MPs E-cadherin(+)MPs ITGB6(+)MPs
Platelet MPs	CD31(+)CD41(+)MPs CD41(+) Annexin V(+)MPs
Eosinophil MPs	EMR1(+)MPs
Mast cell MPs	FcεRI(+)c-kit(+)MPs
Basophil MPs	CD203c(+)c-kit(−)MPs
Erythrocyte MPs	CD235a(+)MPs
Neutrophil MPs	CD66b(+)MPO(+)MPs

**Table 3:** Summary of pros and cons of MPs in human biofluids as biomarkers.

	Pros	Cons
Carrying markers and molecules of parental cells	Possible to evaluate status of cells using combinations of cell-specific markers and molecules on MPs	Sometimes, topology of molecules on MPs may be different from that of parental cells
Detectable in human biofluid samples	Do not have to perform biopsy of tissues	Sometimes, it is difficult to clarify origin of increased MPs in samples (especially blood)
Enable to analyze using flow cytometry	Possible to analyze using small volume of samples EV isolation from samples is not necessary for the analysis	Standard flow cytometry settings and gating strategy have not been established (significantly different from cell analysis)
